# Focus on nursing point-of-care tools: application of a new evaluation rubric

**DOI:** 10.5195/jmla.2022.1257

**Published:** 2022-07-01

**Authors:** Annie Nickum, Emily Johnson-Barlow, Rebecca Raszewski, Ryan Rafferty

**Affiliations:** 1 anicku2@uic.edu, Assistant Professor & Information Services & Liaison Librarian, Library Liaison to the UIC Chicago Nursing Community, University of Illinois Chicago, Chicago, IL.; 2 emj11@uic.edu, Associate Professor & Regional Health Sciences Librarian, Library of the Health Sciences - Peoria, University of Illinois Chicago, Peoria, IL.; 3 raszewr1@uic.edu, Associate Professor & Information Services & Liaison Librarian, Library Liaison to University of Illinois at Chicago Graduate Nursing, University of Illinois Chicago, Chicago, IL.

**Keywords:** point-of-care tool, evaluation rubric, nursing, decision-making

## Abstract

**Objective:**

Point-of-care tools (PoCTs) provide evidence-based information on patient care and procedures at the time of need. Registered nurses have unique practice needs, and many PoCTs are marketed to support their practice. However, there is little reported evidence in the literature about evaluating nursing-focused PoCTs

**Case Presentation:**

The investigators developed a rubric containing evaluation criteria based on content, coverage of nursing topics, transparency of the evidence, user perception, and customization of PoCTs for supporting nursing practice. The investigators selected five PoCTs cited in the literature and of interest to local nursing leadership: ClinicalKey for Nursing, DynaMed, Lippincott's Advisor and Procedures, Nursing Reference Center Plus, and UpToDate. Application of the rubric found Lippincott had the highest coverage of diagnoses, while ClinicalKey for Nursing had strong content focused on interventions and outcomes. Nursing Reference Center Plus provided the most well-rounded coverage of nursing terminology and topics. DynaMed and UpToDate were more transparent with indicating conflict of interest, but both had lower coverage of nursing terminology, content, and care processes.

**Conclusion:**

None of the five PoCTs strongly met all of the evaluated criteria. The rubric developed for this study highlights each PoCT's strengths and weaknesses that can then be used to inform the decision-making process based on priorities and budget. The investigators recommend licensing a nursing PoCT and a PoCT like DynaMed or UpToDate to provide comprehensive, evidence-based, patient care coverage and to meet the diverse information needs of nurses.

## BACKGROUND

Point-of-care tools (PoCTs), each with their own strengths and weaknesses, answer questions at the bedside. Given the cost of these products and the limited budgets of hospitals and libraries, which are best for providing evidence-based nursing care? Health sciences librarians have the requisite knowledge to evaluate the quality of PoCTs for target users [1].

Previous studies evaluating PoCTs have focused on either the medical discipline alone or healthcare in general. There has not been a study comparing PoCTs for nursing information needs. The workflow of nurses differs from other providers; physicians and advanced practice nurses focus more on diagnostics and treatment whereas bedside nurses require the most current information on policies and procedures to support the development and implementation of nursing interventions [2]. Only a PoCT that takes nursing practice into account can address those information needs [3].

This case report presents the development and pilot testing of a rubric to review PoCTs based on the following areas: content, breadth of coverage for nursing, transparency of evidence, user perception, and customization of content.

## CASE PRESENTATION

The University of Illinois at Chicago (UIC) has licensed Nursing Reference Center Plus (NRC+) since 2009. In 2019, the hospital's nursing Advanced Practice and Research Council received a request to review nursing PoCTs to determine which best fit their needs. A librarian who serves as an ad hoc council member brought the request to UIC's nursing librarian team.

The nursing librarians, after reviewing the literature and finding no PoCT rubrics specific to nursing, chose to develop and pilot a rubric to evaluate and compare the PoCTs. Selected for review were three PoCTs that the library already licensed and that were in use by the hospital – DynaMed and NRC+ by EBSCO and UpToDate by Wolters Kluwer. Trials were also obtained for ClinicalKey for Nursing (CK Nursing) by Elsevier and Lippincott Advisor and Procedures (Lippincott) after consulting with nursing leadership.

## METHODS

### Rubric Development

The investigators sought to evaluate the PoCTs from the perspective of which was the best fit for nurses. To create a rubric to do so, the investigators combined criteria from previously developed rubrics [4–7] and then requested input from the nursing council. Criteria included in the final rubric (Appendix 1) focused on content types, breadth of coverage for nursing, transparency of evidence, user perceptions, and customization of content:

- Content types such as continuing education units (CEUs) for registered nurses, patient education materials in multiple languages, and Core Measures from The Joint Commission [8] for benchmarking were deemed crucial for any PoCT with a nursing audience.
- Schurtz and Foster as well as Campbell and Ash [1, 5] had more comprehensive rubrics prompting the addition of customization of content, user perception, and transparency criteria.
- Inspired by Prorok's use of ICD-10 codes to evaluate PoCTs [4], the investigators used standardized nursing terminology to review breadth of coverage. Three terminologies relevant to bedside nurses were chosen: NANDA International Diagnoses, Nursing Intervention Classification (NIC), and Nursing Outcome Classification (NOC) [9-11]. These standardized terminologies are used to create care plans and document nursing care [12]. The investigators randomly selected one term from each of the thirteen domains of NANDA, the eleven domains of NIC, and the eleven domains of NOC for exploration [9–11].
- Campbell's evaluation methods [6] were incorporated into the rubric to determine quality and rigor. The focus on editorial quality–statement of authorship, conflict of interest disclosure, and frequency of updates–influenced the development of the sections on transparency and content types.
- Although Butcher [7] exclusively investigated PoCT apps, her work influenced the transparency and customization of content criteria of whether references were listed, whether authors and peer review were noted, and how often information was updated, as well as app availability.

### Pilot Testing

To test the rubric, four investigators independently extracted the data from the five PoCTs using the rubric in Microsoft Excel from February to April 2020. The investigators marked “yes/no” indicating whether or not they found the criteria in the PoCT. For user perception, a Likert scale was used to rate how information was displayed and ease of use (5=excellent, 1=poor).

For coverage of nursing topics in the rubric, each investigator searched the selected NANDA/NIC/NOC standardized terminology. Searches were constructed at the discretion of the investigator, including techniques such as phrase searching with quotation marks. The investigator reviewed the first ten search results and indicated “yes” in the rubric if the results would be relevant to a bedside nurse.

The investigators identified content types in the PoCTs by reviewing tab and sidebar menus as well as information displayed when searching the coverage of the standardized terminology. Content types were marked as ‘yes' when investigators were able to locate them anywhere within the PoCT.

Transparency of evidence was determined by seeking how evidence-based content was integrated into PoCT summaries as well as disclosure and policy statements on how summaries were produced. The investigators also checked each PoCT for customization of its features with personal accounts and mobile app access.

All extracted data were compiled into a spreadsheet for collective discussion and resolution of disagreements. After all PoCTs were reviewed, the number of items found – or “yes's” – were totaled for the content, transparency, and customization sections. Coverage of nursing topics was calculated by separately totaling the number of NANDA, NIC, and NOC terminologies found by each investigator and dividing by the total number found of each terminology then averaging by four. The user perception results score was calculated by combining the average score of the criteria in that section.

## RESULTS

Results from the rubric pilot testing are reported using descriptive statistics. Tables 1, 2, and 3 (Table 3 – Appendix B) show the number of investigators (0-4) who identified the content, transparency, and customization criteria outlined in the rubric. NANDA/NIC/NOC coverage was broken down by the percentage of relevant results, and user perception was graded on a scale. Complete agreement among all four investigators indicates easily identified information. Disagreement between investigators indicates that not all were able to find the requisite information or deemed their search results relevant.

**Table 1:** Content Types.

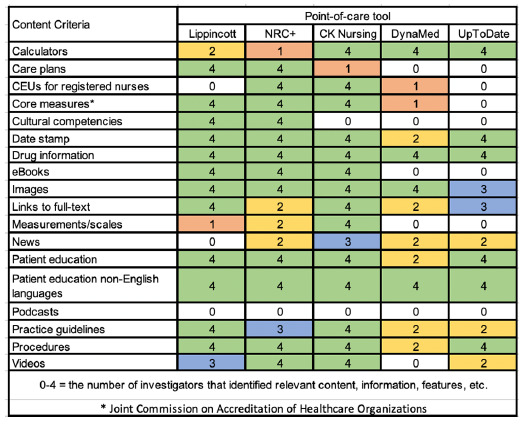

**Table 2:** Transparency Criteria.

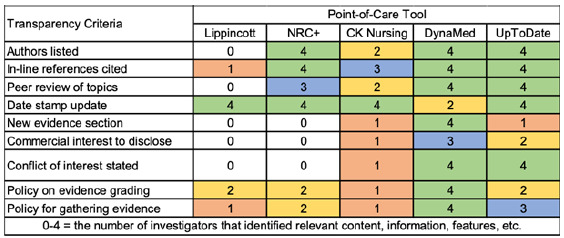

### Content

All PoCTs provided content for nursing (Table 1). However, the PoCTs specifically designed for nursing (CK Nursing, Lippincott, and NRC+) presented a wider range of content relevant to bedside nurses' information needs. CK Nursing ranked highest, with all four investigators finding relevant content for fourteen of eighteen criteria. For Lippincott and NRC+, all four investigators found twelve of the criteria. For DynaMed and UpToDate, there was less agreement between investigators and fewer criteria found. Cultural competencies had minimal coverage in CK Nursing, DynaMed, and UpToDate.

### Coverage of Nursing Terminology

**Figure 1:**
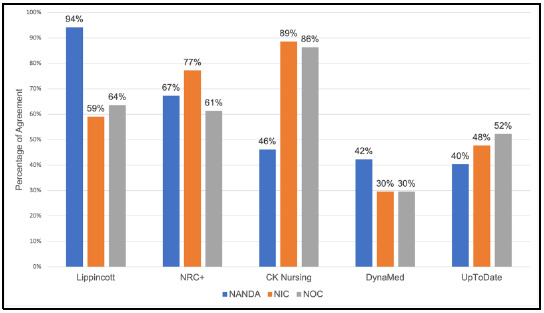
Nursing Terminology.

Figure 1 represents the agreement of relevant results among investigators following the NANDA/NIC/NOC coverage searches. Results were averaged across the four investigators. NRC+ and Lippincott had the best breadth of relevant coverage with all terminologies above 50%. Lippincott showed the most agreement of relevant coverage for diagnoses (NANDA). CK Nursing had higher relevant coverage of information for interventions and outcomes (NIC and NOC) than the other PoCTs. DynaMed and UpToDate had lower relevant coverage.

### Transparency

Transparency criteria assessed content conveying information currency, methods for grading and gathering evidence, and methods for identifying potential bias (Table 2). DynaMed ranked highest, with all four investigators locating seven of nine transparency criteria. For UpToDate, five criteria were discoverable by all investigators. In Lippincott, all investigators found content for only one criterion. Additionally, none found any content related to five of the transparency criteria in Lippincott, and only one located information for two more criteria.

### Customization

Investigator agreement related to customization criteria was lower compared to other rubric categories (Table 3 – Appendix B). Customizable features included saving content, email alerts, CEU tracking, app availability, whether the app could be accessed offline, and if features were present in both the app and web versions. Customizations within personal accounts were not available within all of the PoCTs. Overall, DynaMed and NRC+ ranked higher than the other PoCTs, with three or four investigators finding the customization features for six of the seven criteria. For both CK Nursing and Lippincott, no investigators found features for three of the criteria.

### User Perception

The investigators independently graded user perception on a Likert scale (5=excellent, 1=poor); final results combined all investigators' opinions. Aspects reviewed were ease of navigation, display of information, relevance of information, and ease of searching. DynaMed scored slightly better than the other PoCTs for its content summaries. Overall, NRC+ scored highest on user perception, with 21.75 out of 30 points, followed by Lippincott (20.5 points), DynaMed (19 points), CK Nursing (18.5 points), and UpToDate (17.5 points).

## DISCUSSION

The use of a rubric to evaluate nursing PoCTs offers a transparent process with standardized criteria. Criteria were not only derived from existing literature [4–7], but also customized with nursing terminology. Based on existing rubrics and evaluation tools, as well as the investigators' combined years of experience, we believe the rubric can be used to examine PoCTs' nursing-focused content and coverage. However, there are some factors that could not be built into the rubric, such as cost and overlap of collection coverage. The investigators recommend considering these and other institutional factors in decision-making processes.

The use of a physician-centric approach in presenting information in some of the PoCTs revealed a lack of consideration of how nurses approach patient care. Examples include the minimal relevant nursing subject coverage in DynaMed and UpToDate (as seen in Figure 1) as well as limited contributions by nurse authors in PoCT topics within CK Nursing. Broadly speaking, DynaMed and UpToDate focused more on medical diagnoses while the nursing-focused PoCTs contained nursing procedures and continuity of care.

### Content

Content presentation and delivery varied greatly. The investigators attempted to locate unique nursing-related content within each PoCT, such as care plans, patient handouts in multiple languages, and continuing education. The investigators found that many PoCTs did not crosslink between platforms or related resources to access full text, which could be confusing to end users. Vendors should automatically link licensed content together for findability of full-text or other types of materials. Lippincott combines its proprietary content together well, promoting discoverability between the Advisor and Procedures products, whereas other vendors may require subscribers to ask for linking to be turned on between products. For example, the library had to periodically request that citations from CINAHL be included within NRC+ search results, as the library had separate licenses for NRC+ and CINAHL. Evaluation of the ease of accessing linked resources is a potential addition to the rubric.

The investigators also observed that results did not always prioritize displaying specific content addressing patient care. For example, the results included the NANDA definitions eBook in Lippincott, citations and full-text articles from the CINAHL database within NRC+, and MEDLINE citation records within CK Nursing. While these results were relevant to the search, they did not always support patient care. Search algorithms need improvement in prioritizing actionable PoCT content, such as care plans and procedures, in search results, especially in PoCTs with many content types that support nursing practice.

The inclusion of CEUs in the PoCTs varied, with CK Nursing and NRC+ having CEUs available for registered nurses, while others required additional subscriptions or had no registered nursing CEUs. Although DynaMed and UpToDate include continuing education for nurse practitioners, this does not acknowledge the full scope of nursing practice. Vendors should expand and integrate more CEUs for registered nurses as a core component in PoCTs to continue to strengthen the future of nursing [13,14].

Finally, the investigators sought novel content types as part of the evaluation. One medium examined was podcasts, yet no PoCT produced podcasts. The investigators encourage vendors to expand to this new content medium to provide nurses another opportunity for knowledge attainment.

### Coverage of Nursing Terminology

The investigators' use of NANDA, NIC, and NOC terminologies standardized the process of seeking information related to nursing topics. The investigators observed unbalanced coverage for nursing diagnosis (NANDA), interventions (NIC), and outcomes (NOC). CK Nursing's diagnosis and Lippincott's interventions and outcomes content need further development to support the full nursing care process, from diagnosis to outcome, if they are to be comprehensive. Another challenge was discerning nursing terminology from medical terminology, especially within DynaMed and UpToDate. The investigators acknowledge nurses' usage of these two PoCTs, but their lack of coverage of nursing subject matter is notable.

Finally, the investigators noted inconsistencies in the presentation of the terminologies. The investigators found that some PoCTs presented a NANDA/NIC/NOC topic in the context of a disease or condition summary, while others provided a separate, dedicated summary on that topic. While this presentation of material may be helpful in some contexts such as project planning or research, it required additional work and time scanning results to find what would be applicable for patient care.

### Transparency

The nursing PoCTs did not appear to have conflict of interest policies or statements that were either embedded within their content or available on their products' websites. As evidenced by Table 2, not all investigators were able to locate these materials, which suggests a lack of findability. DynaMed and UpToDate each have conflict of interest policies [15,16] that could be used as exemplars for the nursing PoCTs. Nurses who are authoring or editing content for PoCTs should be required to disclose any potential conflicts of interest. The investigators were also surprised that nursing-related content may not be authored by nurses. For example, some of CK Nursing's materials listed authors who were not nurses. Such an oversight shows a lack of awareness of nurses' expertise and practice. It should additionally be noted that not all content listed authors and that investigators may not have looked at the same entries in each PoCT.

It is critical for healthcare information to be as current as possible. The investigators experienced inconsistencies with how often each PoCT indicated content updates. The investigators also disagreed about how simple it was to identify when a topic was updated. DynaMed was the only PoCT that provided a separate section for updates on its topics. The others that listed a date did not specify which portions of the content had been recently changed. The ability to identify a change in practice standards without having to scan a lengthy document saves nurses' time.

### Customization

The investigators recommend integrating customizable features into each PoCT, such as the ability to add internal notes for procedures and patient education materials or save searches or alerts to help automate processes for nurses. These features need to be user-friendly and more widely available in PoCTs, especially for nurses involved with developing or updating policies and procedures. All PoCTs had an app for mobile devices for both Android and iOS; however, investigators may not have been able to locate or download it offsite. It is also noteworthy that not all apps featured offline capability; this is crucial for units with poor wireless connections.

### User Perception

When creating the rubric, the investigators included criteria focused on user perception. However, in discussing the results, the investigators decided to eliminate this section from the rubric. As librarians, the investigators' perception of usability differs from the PoCTs' main audiences. Furthermore, due to familiarity with and training in various types of evidence-based resources, librarians' views are not representative of healthcare professionals overall. To address this gap, a subsequent study [17] was done to survey nurses' experiences using PoCTs to answer clinical questions.

## LIMITATIONS

The goal of this effort was to develop and pilot test a rubric to evaluate PoCTs for the availability of nursing-focused content. Limitations of the rubric development and pilot testing lie in the difference of the criteria ratings among investigators and biases as librarians.

The rubric was intended to be objective–could the criteria be located within a given PoCT? In the course of pilot testing, the interpretation of the criteria was found to be more subjective than intended. Not all investigators could readily find certain content due to differing approaches for searching PoCTs. For coverage of nursing topics, there was disagreement on whether findings would be sufficient to answer a reference question on the topic. It is reasonable to suppose that if the investigators had difficulty locating relevant information, the average user would as well.

In addition, after developing and pilot testing the rubric, the investigators acknowledged that getting nurses' feedback is essential in evaluating PoCTs. Although librarians can determine if a resource is credible and would meet the end-users' information needs, the resource's main audience can best determine if the resource is user-friendly and worthy of repeated use. Furthermore, it is up to content experts, in this case nurses, to determine relevancy and applicability to information needs.

The investigators reviewed only five of the many PoCTs on the market; two of which were only available through brief trials. Since the other three have been used extensively by the investigators, their familiarity could have influenced their scoring within the rubric. For the customization section, the investigators should have considered accessibility as a criterion. Since some PoCTs include patient education materials, perhaps reviewing how these materials could be customized for patients with disabilities could be included in future criteria. Regarding availability of mobile apps, not all were able to be downloaded offsite. The investigators were working from home due to the COVID-19 pandemic, and those who had not previously downloaded the apps were unable to do so and thus could not evaluate them. Finally, the rubric was neither tested for its validity nor reliability, so further investigation on its future use is warranted.

## CONCLUSIONS

It is imperative that libraries and hospitals work together to make informed decisions about which PoCTs to license. We must move beyond decisions influenced solely by budget and brand recognition. Evaluating products with transparent criteria based on the audience's information needs produces more data to inform the decision-making process.

Overall, none of the PoCTs outperformed the others, and none successfully met all of the rubric criteria. The rubric highlighted each PoCT's strengths and weaknesses to inform the PoCT selection process. For institutions supporting a range of practicing nurses, the investigators recommend including a bedside nursing-focused PoCT and one that would cover advanced nurse practitioners needing more diagnostic and treatment-based information, like DynaMed or UpToDate. The findings of this case report are in line with existing literature: that no one PoCT is best and subscribing to more than one is necessary to meet healthcare professionals' information needs [1,4,6].

Results from this rubric evaluation and a separate survey [17] of nurses' perceptions of using some of the PoCTs were shared with nursing leadership at the University of Illinois Chicago. Nursing leadership did not feel that the findings supported keeping NRC+ and ultimately selected Lippincott Nursing Products. The library canceled its subscription to NRC+ on July 1, 2021. Lippincott Nursing Products was rolled out to the hospital's nursing staff beginning in December 2021.

## Data Availability

Data associated with this article are available in Appendix B. The data will be made available on the authors' institutional repository upon publication.

## SUPPLEMENTAL FILES

**Appendix A:** Rubric Instrument

**Appendix B:** Research Results
