# Supplementary material for: Focus on nursing point-of-care tools: application of a new evaluation rubric
Source: J Med Libr Assoc. 2022 Jul 1;110(3):358–64. doi: 10.5195/jmla.2022.1257 (PMC9782654; doi:10.5195/jmla.2022.1257)
Supplement: Supplementary file 1 — Appendix A: Rubric Instrument [file jmla-110-3-358-s01.pdf]

Appendix A: Evaluation Rubric for the Selection of Nursing Point of Care Tools

**Part One: Content Investigation**				
**PoCT**				
**Reviewer**	**#1**	**#2**	**#3**	**#4**
**Content Found?**	**Yes or No?**			
Procedures				
Practice guidelines				
CE credit for registered nurses				
Patient education				
Other Language Materials?				
eBooks				
Care Plans				
Videos				
Drug information				
Measurements/Scales				
Core Measures				
Images				
Links to full-text				
News				
Calculators				
Podcasts				
Date stamp				
Cultural competencies				

**Part Two: Nursing Terminology Investigation**				
**PoCT**				
**Reviewer**	**#1**	**#2**	**#3**	**#4**
**Terminology Found?**	**Yes or No?**			
**NANDA -13**				
Frail Elderly Syndrome				

Insufficient breast milk production				
Stress urinary incontinence				
Risk of Activity Intolerance				
Labile emotional control				
Readiness for enhanced hope				
Risk of impaired parenting				
Risk of disturbed maternal-fetal dyad				
Grieving				
Spiritual distress				
Delayed surgical recovery				
Chronic Pain				
Risk of delayed development				
**NIC - 11**				
Acid-base management				
Amnioinfusion				
Bladder irrigation				
Cardiac care				
Electrolyte management				
Enteral tube feeding				
Teaching: Infant nutrition				

Communicable disease management				
Shock management				
Medication administration: Subcutaneous				
Environmental management				
**NOC - 11**				
Premenstrual Syndrome severity				
Abuse recovery				
Burn healing				
Child adaptation to hospitalization				
Swallowing status				
Knowledge: Disease process				
Hypertension severity				
Falls occurrence				
Weight loss behavior				
Sensory function				
Mechanical ventilation response: Adult				

**Part Three: Transparency Criteria Investigation**				
**PoCT**				
**Reviewer**	**#1**	**#2**	**#3**	**#4**
**Transparency Criteria Found?**	**Yes or No?**			
In-line references cited				

Authors listed				
Conflict of interest stated				
Commercial interest to disclose				
Frequency of updates				
Date Stamp				
Separate new evidence tab/area of topic				
Peer review of topics				
Policy for gathering evidence/sources - per resource				
Policy on evidence grade/level assigned - per resource/per topic depending on grading system				

**Part Four: Customization Criteria Investigation**					
**PoCT**					
**Reviewer**		**#1**	**#2**	**#3**	**#4**
**Customization Criteria Found?**		**Yes or No?**			
Personalized Account					
	Customization of content				
	Saving content				
	Email Alerts				
	Tracking CEs				
Available as iOS and Android app					

	Offline availability?				
	Features are available on app vs database?				

**Part Five: User Perception of Content Investigation**					
**PoCT**					
**Reviewer**		**#1**	**#2**	**#3**	**#4**
**User Perception of Content**		**Score (1-Low to 5-High)**			
How info was displayed					
	Summary				
	Bullet points				
	Ontology - consistent headers in summary on the nursing care process				
Information displayed is relevant to information need					
Ease of navigation					
Ease of searching database					
