# Supplementary material for: Focus on nursing point-of-care tools: application of a new evaluation rubric
Source: J Med Libr Assoc. 2022 Jul 1;110(3):358–64. doi: 10.5195/jmla.2022.1257 (PMC9782654; doi:10.5195/jmla.2022.1257)
Supplement: Supplementary file 2 — Appendix B: Research Results [file jmla-110-3-358-s02.pdf]

Appendix B

Table 3 Items related to customization

Customizable features	Point-of-care tool				
	CK Nursing	DynaMed	Lippincott	NRC+	UpToDate
Customization of content	0	0	1	1	0
Saving content	4	3	0	4	3
Email alerts	0	3	0	3	0
Tracking CEs	3	3	0	3	2
Available as iOS and Android app	3	4	4	3	4
App offline availability	0	4	2	4	1
Features available on both app and web version	1	4	3	3	3
0-4 = the number of investigators that identified relevant content, information, features, etc.					
